# A Systematic Review of the Wound-Healing Effects of Monoterpenes and Iridoid Derivatives 

**DOI:** 10.3390/molecules19010846

**Published:** 2014-01-13

**Authors:** Rosana S.S. Barreto, Ricardo L.C. Albuquerque-Júnior, Adriano A.S. Araújo, Jackson R.G.S. Almeida, Márcio R.V. Santos, André S. Barreto, Josimari M. DeSantana, Pollyana S. Siqueira-Lima, Jullyana S.S. Quintans, Lucindo J. Quintans-Júnior

**Affiliations:** 1 Department of Physiology, Federal University of Sergipe, São Cristóvão 49.100-000, Sergipe, Brazil; 2 Institute of Technology and Research, Tiradentes University, Aracaju 49.032-490, Sergipe, Brazil; 3 Department of Pharmacy, Federal University of Sergipe, São Cristóvão 49.100-000, Sergipe, Brazil; 4 College of Pharmaceutical Sciences, Federal University of San Francisco Valley, Petrolina 56.304-917, Pernambuco, Brazil

**Keywords:** cicatrix, granulation tissue, terpene, monoterpene, wound-healing, wound closure technique

## Abstract

The search for more effective and lower cost therapeutic approaches for wound healing remains a challenge for modern medicine. In the search for new therapeutic options, plants and their metabolites are a great source of novel biomolecules. Among their constituents, the monoterpenes represent 90% of essential oils, and have a variety of structures with several activities such as antimicrobial, anti-inflammatory, antioxidant and wound healing. Based on that, and also due to the lack of reviews concerning the wound-healing activity of monoterpenes, we performed this systematic review—which provides an overview of their characteristics and mechanisms of action. In this search, the terms “terpenes”, “monoterpenes”, “wound healing” and “wound closure techniques” were used to retrieve articles published in LILACS, PUBMED and EMBASE until May 2013. Seven papers were found concerning the potential wound healing effect of five compouds (three monoterpenes and two iridoid derivatives) in preclinical studies. Among the products used for wound care, the films were the most studied pharmaceutical form. Monoterpenes are a class of compounds of great diversity of biological activities and therapeutic potential. The data reviewed here suggest that monoterpenes, although poorly studied in this context, are promising compounds for the treatment of chronic wound conditions.

## 1. Introduction

Wounds are physical, chemical or thermal injuries that result in an opening or breaking in the integrity of the skin. The continuity of the skin should be restored, and appropriate methods for wound healing are essential for the restoration of disrupted anatomical continuity and disturbed function status of the skin [1].

The acute wound healing process is a complex series of interrelated events that are mediated in its different phases by a wide range of chemically coordinated cellular processes, as well as hormonal influences. It is characterized by a sequence of independent and/or overlapping events [2,3]. The process can be broadly categorized into three or four stages: coagulating phase, inflammatory phase, proliferative phase (formation of granulation tissue and collagen synthesis), and finally the remodeling phase, which ultimately determines the strength and appearance of the healed tissue [4,5,6,7,8].

For centuries, natural products such as medicinal plants have been used to treat a lot of illnesses worldwide, arousing scientific and commercial interests and still playing an important role in the health systems in many developed and developing countries, such as the United States and Brazil, respectively [9,10]. 

Monoterpenes belong to a large and diverse group of chemical compounds named terpenes. They represent a group of naturally-occurring organic compounds. They are the most representative molecules constituting 90% of the essential oils and have a great variety of structures [11], with relevant pharmacological properties such as antimicrobial, anti-inflammatory, antioxidant, antipruritic, hypotensive and analgesic activities [12,13,14,15,16]. Hence, medicinal plants and related compounds have traditionally played an important role in drug discovery and were the basis of most early medicines [17]. Additionally, the usage of techniques and products in wound care allied to substances with anti-inflammatory, antibacterial and antioxidant properties are powerful in the treatment of skin lesions [18]. 

Despite their importance, there are no reviews on the wound-healing potential of monoterpenes. Accordingly, we conducted for the first time a systematic review of the literature to examine and synthesize the literature on monoterpenes, to identify and to evaluate those that assess healing effects in wound-healing animal models.

## 2. Results and Discussion

**Figure 1:**
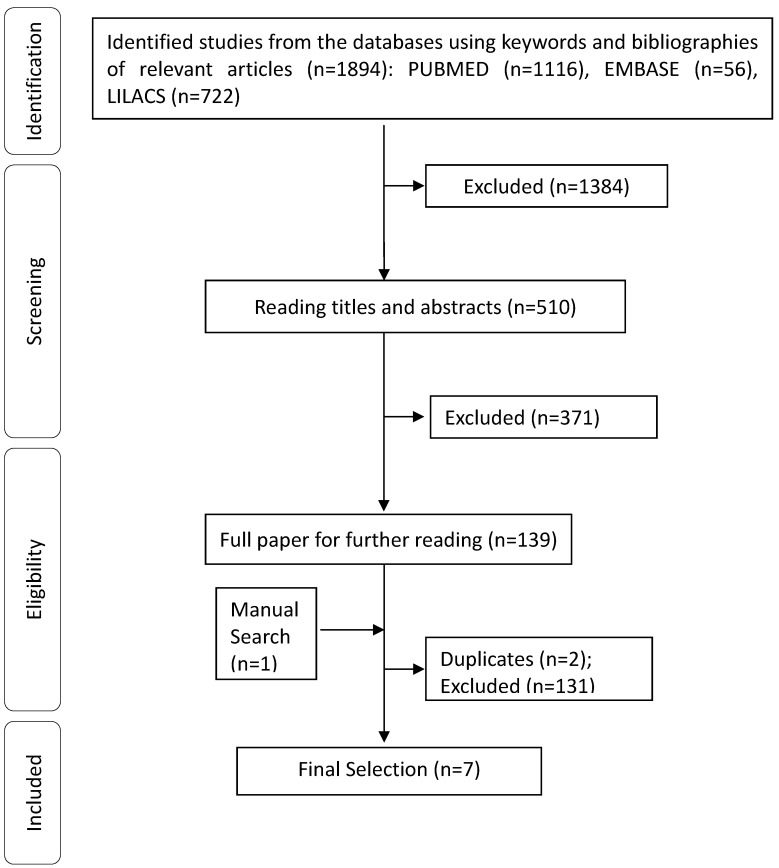
Flowchart of included studies. Studies were excluded according to the following exclusion criteria: studies in humans, studies of mixtures of substances or extracts from plants, review articles, meta-analyses, abstracts, conference proceedings, editorials/letters, case reports.

A total of 1,895 abstracts/citations were identified for preliminary review from electronic and manual searches. The primary search identified 1,894 articles, with 1,116 from PUBMED, 722 from LILACS, 56 from EMBASE and one from manual search. After removal of duplicates and screening for relevant titles and abstracts, a total of 140 articles were submitted for a full-text review. Seven articles met the inclusion and exclusion criteria established. A flow chart illustrating the progress of study selection and article number at each stage is shown (Figure 1).

From seven final selected studies, most of that research was conducted in China (43%), Korea (29%), Brazil (14%) and Peru (14%). Regarding the pharmaceutical form of the products used for wound healing presented in the selected studies (Table 1), bioactive films (38%) were the most used, followed by ointments (25%) and solutions (25%) and finally suspensions (12%).

**Table 1:** Characteristics of included studies.

Authors, year, Country	Substance(s)	Animals	Doses, Concetration or Quantity	Pharmaceutical dosage forms	Model	Valued Parameter Settings		Results and Mechanisms
						Macroscopic	Microscopic	
Mai L.M. *et al.*, 2003, China	Borneol	Adult male *Sprangue-Dawley* rats	4.5% and 0.7%	Vaselin-based ointment	Excision wound	Basic physiological conditions (body length, weight, food eaten, water intake); Wound areas measured by slide calipers and photographed	Histological observation (HE) (skin appendages and collagen fibers quality); Measurement of the thinckness of the granulation tissue and newly formed epithelium	This study found that the combination of Bismuth subgallate (BS) and Borneol (BO) and Vaseline had a synergistic effect in accelerating wound closure. All results were associated with the BS, while nothing about BO was reported. However, the precise mechanism of the drugs remains unclear and further work is necessary to study whether macrophages could secret growth factor to accelerate wound healing.
Riella K.R. *et al.*, 2012, Brazil	Thymol	Adult male and female *Wistar* rats	1.2 mg	Collagen-based films	Excision wound	Wound contraction rates by digital caliper	Inflammatory response and profile inflammatory (HE); Collagen deposition (Picrosirius)	The modulation of the leukocyte influx by thymol was associated to increased levels of macrophage migration inhibitory factor (MIF) in central nervous system; The improved on granulation tissue by collagen-based containing thymol (COLTHY) films founded was associated to anti-inflammatory properties of thymol; Improvement in the replacement and arrangement by COLTHY was associated to modulatory effect on the flbroblast metabolism and collagen synthesis and the thymol able to enhance the flbroblasts growth *in vitro*.
Zhang K. *et al.*, 2010, China	Genipin	*Sprague–Dawley* rats	50 mg/mL	Silk fibroin/ hydroxybutyl chitosan	Excision wound	---------	Histological examination of the inflammatory response; epithelization; proliferation of fibroblasts and collagen proliferation; blood vessels migration.	Greater proliferation of fibroblasts was observed in the nanofibers that was associated to a genipin crosslinked; Fibroblasts cells had greater proliferation and arranged in better order, densely in nanofibers that was too associated to a genipin crosslinked.
Villegas L.F. *et al.*, 2001, Peru	α-Terpineol	Male mice	0.05 mL	Suspension	Incision wound	Tensile strength	---------	Epi-α-bisabolol, α-bisabolol and α-terpineol showed significant *in vivo* cicatrizant activity and did not have a significant effect on increasing cell migration. The mechanisms is not shown.
Chang W.H. *et al.*, 2003, China	Genipin	Male *Wistar* rats	---------	Wound dressing Membranes	Excision wound	Basic physiological conditions (infection)	Histological examination of the inflammatory response; epithelization and granulation tissue formation.	Genipin-crosslinked dressing membrane showed lower inflammatory reaction in the wound that may be due to the lower toxicity of its remaining residuesGenipin-crosslinked dressing membrane promotes early re-epithelialization, but mechanisms is not shown .
Lee S.W. *et al.*, 1999, Republic of Korea	Aucubin	Male rats	0.1%	Solution and based ointment	Incision wound	---------	---------	---------

In the selected articles, the wound models used to study the wound healing included excision and incision wound model. However, the main model used was the excision wound model (83%). In most of selected studies, both macroscopic and microscopic features were evaluated. A total of 56% selected searches evaluated tissue morphology aspects involved in the wound healing process.

Concerning the mechanisms of action involved in the wound healing proposed for different monoterpenes, the ones suggested were antimicrobial activity (inhibits RNA and protein biosynthesis of microorganisms), anti-inflammatory activity (decreases the amount of IL-6 and TNF-α production in mast cells, inhibits the release of LTC4 and has an effect on the release of TXB2); antioxidant activity (photoprotective effects and oxidative stress by inhibiting UVB-induced free radical production); low-toxicity characteristics, macrophage migration inhibitory factor (MIF) and fibroblast growth effects.

In this study, China was the country with the largest number of studies on the healing effects of monoterpenes. The use of plants for medicinal purposes to treat, cure and prevent diseases is one of the oldest forms of medical practice of Humanity [19]. In particular, Traditional Chinese Medicines (TCM) are composed by various combinations of medical plants and have been used as natural remedies for thousands of years [20].

Medicinal plants are the primary sources of many small molecule drugs and herbal products [21,22]. Several recent publications reiterate the importance of natural products as a source of drugs [23,24]. In this context, the advent of modern technologies has boosted medicinal plants as a highly valuable commodity in the patent market. Many developed and developing countries are actively engaged in the biomining of medicinal plants for therapeutically precious and biologically active phytochemicals [25].

According to the World Conservation Monitoring Centre (WCMC) of United Nations Environment Program (UNEP), China was identified as one of the largest mega-biodiversity countries [25]. According to Ravenhill, China is one of the largest countries in Asia, which have the richest arrays of registered and relatively well-known medicinal plants [26]. In addition, medicinal plants have been used in developing countries for thousands of years. The World Health Organization (WHO) estimated that 70%–80% of the population living in developing nations depends on traditional healthcare systems for primary healthcare [27]. Besides, in China about 40% of the total medicinal consumption is attributed to traditional medicines [27]. 

Brazil is one of the countries with the largest biodiversity in the planet and it is associated with an extensive ethical and cultural diversity (Indigenous, African and European) that traditionally uses natural products. It also presents social and economical characteristics that typify it as a developing country, where 80% of the population depends on the use of plants for the primary health care [28,29]. 

Despite the therapeutic potential of medicinal plants and their compounds, the great biodiversity and also the ethnic and cultural aspects of developing countries such as China and Brazil, few studies were found regarding the wound-healing effects of monoterpenes. For this study, only isolated monoterpenes were included, due to the fact that they provide structural molds for obtaining synthetic substances and also because they are considered as sources for drug development. Furthermore, they can be used as tools to identify mechanisms of action [30].

The healing process can be accelerated and enhanced by the use of wound care techniques and products [31,32]. In this review, it was observed that, among the products used for the wound care, the films were the most studied pharmaceutical form. The use of liquid dosage form provides the advantage of studying the action of the isolated compounds. However, the major problem is the short residence times on the wound site, especially where there is a measurable degree of wound fluid exuding [33].

It might also be noted that the bioactive films were studied in the most current research. Currently, it has been shown that wound healing becomes rapid and successful when a warm moist environment around the wound is provided. Unlike the solutions, recently the modern dressings have been developed with features to retain and create this great environment playing an active role in wound healing [33]. 

Moreover, the dressings-based biomaterials, for being part of the matrix of natural tissue, are biocompatible at the toxicological point, biodegradable and are able to permeate active ingredients such as antimicrobial agents or growth factors [34,35].

Wounds are heterogeneous, and the wound-healing process is multifactorial, and influenced by many extrinsic and intrinsic factors. In order to obtain new knowledge of the complexity of this process or substances effects, the use of animal models is required [36]. 

More specific human chronic wound treatments are absent, in a large part due to the lack of knowledge of the molecular abnormalities within the wound that prevent healing. Research is hindered by the absence of an easily reproducible animal model that mimics the human chronic wound state [37,38]. Currently, the chronic wound models described (and that represent the best available at present) are far from ideal [38].

In the present study, the excisional wound model was the most used. This is an acute wound model whose great advantage is the rapid introduction of injury and a relatively rapid course, besides being a wound model of easy and inexpensive execution when compared to chronic wound models [38,39].

Furthermore, the excisional wound model involves the removal of a significant volume of the target tissue, and the filling of the void created allows greater amount of material. The excision site can be harvested or biopsied to obtain cells, tissue, RNA, exudates, and histological specimens that have a wider cross-sectional area and volume when compared to incisional wound. This is suitable for *in situ* techniques or biomechanical strength (tensile strength) [39,40]. 

Concerning the evaluated parameters, the analysis of the kinetics of biological events in response to pharmacological substances is crucial for the development of effective therapeutic products able to stimulate wound healing [40]. This review shows that no study prioritized the molecular biology assays.

Monoterpenes or monoterpenoids are compounds with a core of 10 carbons. They are cyclized and oxidized in a variety of ways. Due to the low molecular weight, many of them exist in the form of essential oils [11]. A type of monoterpenes, the iridoids, is derived from geraniol. They are different from the similarly-named iridals (triterpenes). A subclass of iridoid, the iridoid glycosides and glucosides are compounds that include a glycoside or glucoside, respectively, moiety, usually found at the C-1 position.

In the present study, articles with the following monoterpenes were selected, including types and their subclasses: borneol, thymol, α-terpineol, genipin and aucubin. According to the scientific literature, such compounds possess a range of biological activities that may be directly or indirectly related to wound-healing effects. 

**Figure 2:**
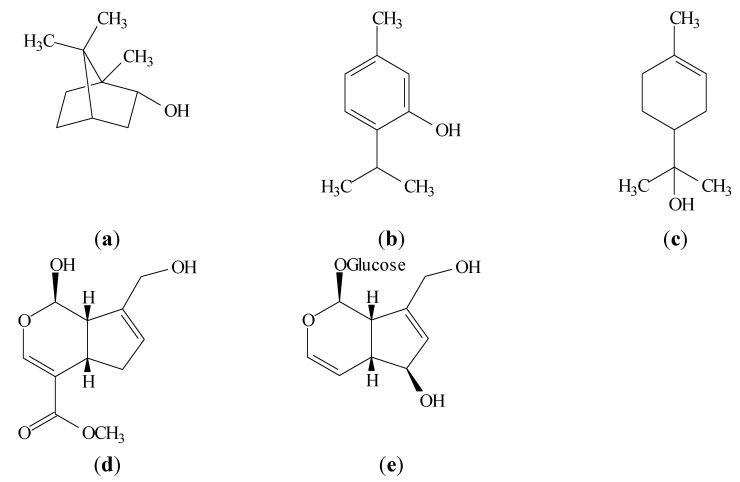
Structural formulae of (**a**) (−)-borneol, (**b**) thymol, (**c**) α-terpineol, (**d**) genipin and (**e**) aucubin.

Borneol is a bicyclic monoterpenoid alcohol (Figure 2a). Borneol has shown effects such as antibiotic activity [41], wound-healing activity [42], anti-inflammatory activity by reducing leukocyte migration [43], anti-fibrosis activity by decreasing the fibroblasts growth, inhibiting collagen production, decreasing MMP-2 activity and inhibiting TIMP-1 production [44]. It showed no cytotoxicity [44], radical scavenging properties [12,45] and immunomodulatory effects [46]. This monoterpene was able to suppress the proinflammatory cytokine (IL-1β and IL-6) mRNA expression and act as bioactive material in the cellular signal transduction system [47]. It shows antibacterial activity and inhibitory effects on several Gram (−)ve and Gram (+)ve pathogenic microorganisms [48,49], antifungal activity [12,48,50,51,52], antioxidant activity by reducing intracellular reactive oxygen species (ROS) generation and attenuating the elevation of nitric oxide (NO), the increase of inducible nitric oxide synthase (iNOS) enzymatic activity and the upregulation of iNOS expression [53]. Borneol blocked NF-κB p65 nuclear translocation [53] and was shown to be a mast cell membrane stabilizer [54]. Finally, anti-inflammatory property was shown through fewer ICAM-1 positive vessels, IL-1β positive cells, TNF-α positive cells and number of neutrophils [55].

Thymol is a monoterpenoid phenol (Figure 2b) which exhibits multiple biological activities. Studies show that thymol modulates prostaglandin synthesis [56], it has anti-inflammatory effect in human neutrophils incubated [57] and beneficial effects on the antioxidant status by the influenced on docosahexaenoic acid (DHA) concentration [58]. Thymol prevented autoxidation of lipids [59] and the formation of toxic products through the action of reactive nitrogen species [60]. It exhibits antimicrobial activity [57,59,61,62,63] and wound-healing activity [64]. Thymol is able to increase the levels of macrophage migration inhibitory factor (MIF) in central nervous system [65], enhance the *in vitro* fibroblast growth [66] and interfere with elastase activity as evidenced by the reduced release of this proteinase by human neutrophils stimulated with the synthetic chemotactic peptide *N*-formyl-methionyl-leucyl-phenylalanine (fMLP) [57]. It effectively inhibited COX-1 [67], inhibited inducible lymphocyte proliferation [68] and showed anti-inflammatory effects through the reduction of the edema, inhibition of MPO activity and decreased leukocyte influx [64].

α-Terpineol is a monoterpenoid alcohol (Figure 2c) relatively non-toxic which is present in the essential oils of several species [69,70]. This monoterpene presented wound healing effect [69] and anti-inflammatory activity by inhibiting the COX enzyme and IL production [71,72]. α-Terpineol is also an NF-κB inhibitor and promotes down-regulation of IL-1β expression [73] and IL-6 formation [74]. Futhermore, the power in the reduction of TNF-α and NO production was demonstrated [75]. In addition, α-terpineol showed selective inhibition of ovine COX-2 activity [72], inhibited the neutrophil influx [75], exhibited strong antimicrobial activity [76] and antifungal effects [77].

Genipin is an iridoid compound (Figure 2d) and an alternative natural crosslinking agent [78,79,80,81,82]. It has shown ability to form biocompatible and stable crosslinked products and showed low cytotoxicity [83]. Moreover, it has been proved that genipin has anti-inflammatory [84], wound healing [81,82] and anti-oxidative effects [85] and abilities of inhibiting lipid peroxidation and production of nitrogen monoxide (NO) [86]. Additionally, genipin can increase the mitochondrial membrane potential [87], increase the ATP levels and close KATP channels [87] and stimulate insulin secretion [87]. Finally, studies showed that genipin suppress the alpha-TN4 lens epithelial cells and subconjunctival fibroblast migration induced by TGF-b [88,89].

Aucubin is an iridoid glycoside (Figure 2e) with a variety of pharmacological effects, such as antimicrobial [90,91,92], anti-inflammatory [93,94], dermal wound healing [95,96] and *in vitro* antioxidative capacity [97]. In addition, aucubin showed inhibition of RNA and protein biosyntheses [91,95,98,99,100]. Futher, aucubin inhibits TNF-α-induced secretion and mRNA synthesis including PAI-1, MCP-1, and IL-6 [101]. Furthermore, investigation revealed that aucubin suppressed extracellular signal-regulated kinase (ERK) activation [102], inhibitory kappa Bα (IκBα) degradation [102], and subsequent nuclear factor kappa B (NF-κB) activation [102]. Finally, aucubin was considered as a promising chemopreventive agent and was devoid of any cytotoxic activity [103,104,105].

## 3. Experimental

The present systematic review was conducted according to the guidelines for Transparent Reporting of Systematic Reviews and Meta-Analyses (PRISMA statement) [106]. 

### 3.1. Search Strategy

Three databases (Internet sources) were used to search for appropriate papers that fulfilled the study purpose. Those included the National Library of Medicine, Washington, DC, USA (MEDLINE-PubMed), Excerpta Medical Database by Elsevier (EMBASE), and Latin American and Caribbean Health Sciences (LILACS), using different combinations of the following keywords: wound healing, wound closure techniques, cicatrix, granulation tissue, monoterpenes and terpenes. The databases were searched for studies conducted in the period up to and including May 2013. The structured search strategy was designed to include any published paper that evaluated a wound healing to identify those that show potential therapeutic value. Citations were manually limited to animal studies. Additional papers were included in our study after analyses of all references from the selected articles. We did not contact investigators, nor did we attempt to identify unpublished data. 

### 3.2. Study Selection

All electronic search titles, selected abstracts, and full-text articles were independently reviewed by a minimum of two reviewers (R.S.S.B., A.S.B. and L.J.Q.J.). Disagreements on study inclusion/exclusion were resolved with the reach of a consensus. The following inclusion criteria were applied: wound-healing studies, and the use of monoterpenes isolated or not isolated from medicinal plants (natural or synthetic product) for treatment. Studies were excluded according to the following exclusion criteria: studies in humans, studies of mixtures of substances or extracts from plants, review articles, meta-analyses, abstracts, conference proceedings, editorials/letters, case reports (Figure 1).

### 3.3. Data Extraction

Data were extracted by one reviewer using standardized forms and were checked by a second reviewer. Extracted information included data regarding the substance, animal models, dosages and concentrations, dosage form, evaluated parameters, results and proposed mechanisms of action.

## 4. Conclusions

For more than a decade, researchers have studied the wound-healing potential of monoterpenes through *in vivo* and *in vitro* assays. Therefore, as in Nature there are about 20,000 known different terpene metabolites [107], this superfamily, of which the monoterpenes are a part, still offers a great opportunity of new discoveries for this application. Nevertheless, this review described the study of only five monoterpenes or types of monoterpenes in models of wound healing in animals. In summary, it can be concluded that, although there are some studies about the wound-healing effects of monoterpenes, a class of compounds of great diversity of biological activities and therapeutic potential, they have been little studied for the treatment of wounds, which occurs especially in developing countries that have a wide biodiversity and tradition in the use of natural products such as Brazil.

Moreover, of those, every evaluated monoterpenes showed wound-healing effects. The anti-inflammatory action of monoterpenes is often related and correlated to wound-healing effect. However, further studies are required to better understand these mechanisms. All these findings make the monoterpenes a great potential source of compounds for the development of new drugs for the treatment of various pathological processes that afflict humanity, including chronic wound conditions.
